# Advancing and strengthening the study of social networks in community-level dissemination and implementation research: A narrative review

**DOI:** 10.1017/cts.2024.614

**Published:** 2024-10-28

**Authors:** Ariella R. Korn, Jennifer L. Cruz, Natalie R. Smith, Rebekah R. Jacob, Megan Carney, Wallis Slater, Shoba Ramanadhan

**Affiliations:** 1 Behavioral and Policy Sciences Department, RAND, Boston, MA, USA; 2 Department of Social and Behavioral Sciences, Harvard T.H. Chan School of Public Health, Boston, MA, USA; 3 Prevention Research Center, Brown School Washington University in St Louis, St. Louis, MO, USA

**Keywords:** Social networks, community-level, theories, models and frameworks, implementation strategies, implementation science

## Abstract

The dissemination and implementation (D&I) of evidence at the community level is critical to improve health and advance health equity. Social networks are considered essential to D&I efforts, but there lacks clarity regarding how best to study and leverage networks. We examined networks in community-level D&I frameworks to characterize the range of network actors, activities, and change approaches. We conducted a narrative review of 66 frameworks. Among frameworks that explicitly addressed networks – that is, elaborated on network characteristics, structure, and/or activities – we extracted and synthesized network concepts using descriptive statistics and narrative summaries. A total of 24 (36%) frameworks explicitly addressed networks. Commonly included actors were implementers, adopters/decision-makers, innovation developers, implementation support professionals, and innovation recipients. Network activities included the exchange of resources, knowledge, trust, and norms. Most network-explicit frameworks characterized ties within and across organizations and considered element(s) of network structure – for example, size, centrality, and density. The most common network change strategy was identifying individuals to champion D&I efforts. We discuss opportunities to expand network inquiry in D&I science, including understanding networks as implementation determinants, leveraging network change approaches as implementation strategies, and exploring network change as an implementation outcome.

## Introduction

Improving the quality and equitable delivery of evidence-based interventions, practices, programs, policies, and other health innovations (herein referred to collectively as EBIs) is critical for improving population health and addressing health inequities [1]. The field of dissemination and implementation (D&I) science is particularly focused on how EBIs for health are delivered in real-world settings. Dissemination refers to the targeted distribution and communication of information, intervention materials, and evidence to specific audiences, whereas implementation is the adoption and integration of EBIs into routine practice or care [2]. Key settings for improving the D&I of EBIs are within communities. Community-level action (i.e., change efforts in community and public health organizations and settings) to improve EBI access and delivery is vital [3], given that opportunities to live a healthy life are largely determined by the communities in which individuals live, work, worship, and play [4]. Community-level action may seek to address barriers to the D&I of EBIs and/or leverage factors that facilitate EBI spread and delivery in community settings (e.g., through coalition building, intervention tailoring, and use of local opinion leaders, among other strategies) [5,6].

One opportunity to support community-level action comes from attending to the social processes underlying the D&I of EBIs. Social processes, rooted in the relationships and interactions among a range of actors, play a major role in D&I success. There are many actors involved in community-level efforts, including organizations, decision-makers (e.g., policymakers and community leaders), individuals involved in disseminating and implementing EBIs (e.g., practitioners, educators, clinicians, and implementation support professionals), and EBI recipients (e.g., students, patients, and families) [7,8]. In the context of D&I efforts, actors’ relationships within networks facilitate the exchange of information and resources, influence, advice sharing, mentoring, capacity building, and collaboration, among other social processes [3,7].

To study the network structure of D&I actors’ relationships within communities and how these relationships influence D&I behaviors and social processes, we can utilize social network analysis (SNA) [9,10]. SNA focuses “nodes,” which can be individuals or organizations, and the relationships between them (referred to as “ties”). Both nodes and ties can be defined by characteristics. For example, if nodes are individual people, they can be characterized by sociodemographic information. Ties can be characterized by the strength or directionality of the relationship (e.g., how often two individuals are in contact with each other). SNA can inform D&I efforts by helping to identify and characterize efficient pathways by which EBIs can be spread and scaled, explore social influences on decision-making processes, predict and explain tie formation patterns, examine connections between network characteristics and attitudes held by network members, and more [7,11]. For example, Palinkas and colleagues utilized SNA to examine the structure of a network of social service organizations in relation to decision-making around adoption (or rejection) of a novel EBI. Their mixed-methods evaluation offered (a) a holistic view of the patterning of connections within the network and the impact of that patterning, and (b) an understanding of what flowed across network connections [12]. Unlike conventional analytic approaches, which treat individuals as independent actors, SNA offered the opportunity to leverage the interconnected nature of network members in the analysis.

SNA provides a foundational understanding of networks’ structure, activities, and performance; this knowledge can be used to consider how to strengthen the effectiveness of networks for D&I efforts [7,9,13,14]. Valente’s 2012 publication in *Science* on “network interventions” highlighted how the structure and characteristics of social networks can be leveraged to initiate, change, or maintain network behavior to positively impact health. Valente described four categories of interventions: identifying individuals to act as change agents (e.g., opinion leaders, champions, and individuals who bridge different groups); delivering an intervention to segments or subgroups in a network based on relationship structure (e.g., clusters or cliques) or attributes (e.g., sociodemographic characteristics); inducing or prompting change using network structure (e.g., word-of-mouth tactics for disseminating information, snowballing); and altering or changing the network structure itself by adding or removing people, groups, or connections [10]. Such network change interventions allow for purposeful adaptations that improve network function and outcomes. Despite the potential of these interventions, much of the work in the field of public health has focused on network interventions as applied to health behavior change interventions [15–17], rather than the using network interventions within D&I research.

In D&I research, network interventions offer a way to affect systems change and improve the utilization and dissemination of EBIs. Indeed, network change interventions are similar in concept to several established implementation strategies (methods and techniques to promote the implementation and sustainability of EBIs). For example, the Expert Recommendations for Implementing Change includes a category of “developing stakeholder interrelationships,” which includes specific implementation strategies like building a coalition, creating a learning collaborative, developing new clinical teams, identifying champions and early adopters, and promoting network weaving [5]. Similarly, in the taxonomy of D&I strategies offered by Leeman and colleagues [6], the fundamental emphasis on the delivery system, support system, and synthesis/translation system inherently supports incorporating SNA perspectives. Further, a 2023 scoping review explicitly sought to identify network change strategies that could serve as implementation strategies and offers additional insight. Based on a review of 53 studies, Bunger and colleagues proposed a multilevel typology of network interventions that includes (a) *actors* (e.g., by changing an actor’s prominence in the network or motivation to connect with others), (b) *relationship* creation, strengthening, or dissolution (e.g., through incentives or training), and (c) *context*, defined as changing the environment, creating groups, and changing network composition or membership [18].

SNA is not new to D&I research [19,20], and networks were foundational to the Diffusion of Innovations theory [21] upon which much of D&I is grounded. However, much of the literature connecting SNA and D&I focuses on the impact of network connections on organizational functioning (e.g., trust) rather than the spread or integration of EBIs [11]. Additionally, few extant D&I network studies focus on community-level action to help improve the D&I of EBIs [11]. To advance this work, it is critical to incorporate a solid theoretical foundation to strengthen how the field understands and uses implementation strategies that target network change processes [18]. There are many D&I theories, models, and frameworks (TMFs), but it is unclear whether these TMFs provide sufficient specification regarding how to identify and leverage networks in D&I strategies aiming to improve the reach, utilization, spread, and sustainability of EBIs.

This is a lost opportunity, particularly given the importance of networks and relationships in D&I science and practice. Therefore, the objective of this research was to (1) examine the *explicit* presentation of social network concepts in D&I TMFs (i.e., concepts that address the structure of relationships and how these relationships influence behavior and social processes) that include community-level action and (2) identify opportunities to advance the study, specification, and use of networks in D&I efforts. This work is an important step toward moving beyond reporting on networks as part of context to strategically intervening on networks.

## Methods

We conducted a narrative review of network concepts in D&I TMFs that target community-level change. Compared to other review methodologies, a narrative review aligns with our study objective as an approach to explore, identify, and descriptively summarize a broad range of concepts at the intersection of networks, communities, and D&I TMFs.

### Selection of TMFs and TMF publications

The current work leverages an extant review published in 2021 by Pinto et al. of 74 community-level TMFs that describe aspects of community engagement in D&I research and practice – including communication, partnership exchange, community capacity building, leadership, and collaboration [3]. Given (a) our similar focus on community-level change in D&I efforts and (b) the inherent role of social networks in community engagement processes (e.g., forging new collaborations), we built on the Pinto et al. review by bringing an explicit attention to networks in D&I TMFs. We used the collaborative, web-based Covidence platform [22] to manage the database of TMF publications cited in the Pinto et al. review, including grouping multiple publications for a single TMF in the same record to streamline data extraction. One TMF in the Pinto et al. review was listed in duplicate and merged for the current study. We contacted authors of original articles when necessary (i.e., to request full-text articles that we could not access via institutional subscriptions). From the existing Pinto et al. sample, we selected 66 TMFs for the current review, each with at least one corresponding publication, that met the following inclusion criteria (Supplementary File 1): relevant to community-level dissemination and/or implementation efforts (n = 5 excluded); available as full-text articles (n = 1 excluded); and available in English (n = 1 excluded). Potential exclusions from the Pinto et al. list were discussed as a team.

### Data extraction of network concepts

We iteratively developed our data extraction template. We first conducted a preliminary review of nine commonly used TMFs in D&I science [21,23–31] and noted network constructs and relevant information. The network information gathered in this preliminary review drove our data extraction template, which we pilot-tested and iteratively refined with six TMFs [23,24,32–36]. Data extraction focused on descriptions of the TMF (i.e., the article narrative explaining the features and constructs of the TMF, in addition to the TMF figure or visual if applicable). Information in the included articles that described formative research, case studies, examples, and applications of the TMF were excluded from the data extraction process.

Using Covidence, two coders independently completed data extraction, with a third coder conducting consensus and the team reviewing any outstanding disagreements. For each included TMF, we extracted the TMF title and the year the TMF was first published. In collecting network information, our first step was to determine whether the TMF explicitly addressed networks – that is, the TMF elaborated on network characteristics, network structure, and/or network activities beyond simply acknowledging that relationships or partnerships are important for D&I efforts. For example, we determined that the Conceptual Model for the Diffusion of Innovations in Service Organizations is a “network-explicit TMF” because it includes several constructs describing relationship characteristics and their network structure (e.g., homophily, decentralization) [26]. Conversely, while the Dynamic Sustainability Framework emphasizes the need for stakeholder partnerships to maintain the use of EBIs [27], this TMF does not offer further information about network concepts and therefore we did not determine it to be network-explicit. Other examples of relational or social concepts that could be inferred from TMFs – but would not qualify as network-explicit without further network detail – include considerations of communication, social influence, sharing values and goals, social support, and implementation facilitation.

Table 1 defines key network concepts applicable to our TMF data extraction. For each network-explicit TMF, we extracted the following information:

**Table 1.** Network concepts extracted from network-explicit TMFs

Concept	Definition [7,9,10]	Application for TMF data extraction
Nodes	Individuals and/or groups in the network	Actors directly or indirectly involved in D&I efforts, including EBI recipients, implementers, implementation support professionals, decision-makers, policymakers, funders, and EBI developers Implementing or disseminating organizations or agencies Community-based organizations or faith-based organizations involved in D&I
Ties	Relationships or connections between nodes that provide the foundation of network structure	Presence of relationships within (intra-) and/or across (inter) organizations that are directly or indirectly involved with D&I efforts
Tie type and purpose	Activity or function of ties (e.g., information sharing or advice sharing)	Description of what is built or exchanged across network ties and/or the outcomes of network activity in support of D&I efforts
Tie characteristics	Example tie characteristics: Extent that ties are “strong” or “weak” based on their function (e.g., for a communication tie, the distinction between frequent interaction vs. infrequent interaction) Tie homophily: similarity of connected nodes based on a given attribute (e.g., role) [61]	Description of how ties are characterized that may promote or hinder D&I efforts
Structural characteristics	Example structural characteristics: Network size Centrality: extent of a node’s connectedness Bridging: nodes connecting network subgroups Density: extent of overall network connectivity or cohesion	Description of how network structure is characterized that may promote or hinder D&I efforts
Network change interventions	Approaches to change network structure or network characteristics to initiate, change, or maintain behavior	Inferences drawn from TMFs about how to change network structure or characteristics to support D&I efforts, informed by Valente’s four categories of network interventions: [10] Identifying individuals to act as change agents (e.g., leaders of faith-based organizations or community champions) Delivering an intervention (e.g., training, technical assistance) to network subgroups Inducing or prompting change using network structure (e.g., using snowball techniques to reach EBI recipients) Altering or changing network structure (e.g., adding ties between organizations to improve EBI dissemination)

D&I = dissemination and implementation; EBI = evidence-based intervention; TMFs = theories, models, and frameworks.

- Types of network actors and organizations (e.g., recipients, implementers);
- Whether networks occur within and/or across organization(s) involved with D&I efforts;
- What is built or exchanged across the network (e.g., information, innovation) and/or the outcomes of network activity (e.g., trust, social capital);
- How relationships are characterized (e.g., based on tie strength or weakness);
- How network structure is considered (e.g., size, centrality);
- Guided by Valente’s taxonomy of network change interventions [10], ways in which the TMF proposes ways to change the structure or characteristics of networks.

A copy of our Covidence data extraction template is available in Supplementary File 2.

### Data analysis

Among the network-explicit TMFs, we synthesized network concepts using descriptive statistics and narrative summaries.

## Results

Of the 66 TMFs in our review sample, about one-third (n = 24; 36%) included explicit attention to social networks [21,23,24,26,29,32,35–60] (Supplementary File 3). These network-explicit TMFs were initially published between 1969 and 2016. Below we summarize our findings according to network concepts outlined in Table 1. We describe example TMFs to demonstrate the range of network considerations.

### Nodes

As shown in Table 2, the 24 network-explicit TMFs included a diverse range of actors. Commonly included actors were implementers (n = 22), adopters or decision-makers (n = 22), innovation developers (n = 18), implementation support professionals (n = 14), and innovation recipients (n = 12). Fewer frameworks discussed the network roles of policymakers (n = 11) and funders (n = 5). At the organizational level, most TMFs described implementing or disseminating agencies (n = 20), and more specifically, community-based, or faith-based organizations (n = 16) involved in D&I efforts.

**Table 2.** Types of actors in network-explicit TMFs (n = 24)

Type of actors	# TMFs	%
***Individual actors***		
Implementers (e.g., providers, practitioners, service-delivery persons)	22	91.7
Adopters or decision-makers (e.g., organizational leaders)	22	91.7
Researchers or innovation developers	18	75.0
Implementation support professionals (e.g., technical assistance providers)	14	58.3
Innovation recipients (i.e., people getting the health service of interest)	12	50.0
Policymakers	11	45.8
Client interpersonal network members (e.g., family members or peers)	5	20.8
Funders or payors	5	20.8
Other: employees within an organization (role not otherwise specified)	1	4.2
***Organizational actors***		
Implementing or disseminating agencies or organizations	20	83.3
Community-based or faith-based organizations	16	66.7
Other: advocacy groups	2	8.3
Other: media	1	4.2

TMFs = theories, models, and frameworks.

*Note:* Types of actors are listed in descending order based on frequency. Multiple selections were allowed.

### Ties within and across organizations

One TMF (4%) addressed intra-organizational networks only, three TMFs (13%) addressed inter-organizational networks only, and 20 TMFs (82%) addressed both (Table 3). Some TMFs such as the Consolidated Framework for Implementation Research (CFIR) [29,39] and Exploration, Preparation, Implementation, and Sustainment (EPIS) framework [23,24] describe intra- and inter-organizational network structure according to the inner and outer context for implementation. Notably, the Conceptual Model for the Diffusion of Innovations in Service Organizations emphasizes the importance of networks at multiple levels and phases of implementation, including internal and external networks that support knowledge sharing as a “system antecedent for innovation,” networks of the adopter within the “user system” where implementation occurs, and inter-organizational networks in the outer context that influence norms and the broader implementation environment [26].

**Table 3.** Characteristics of network-explicit TMFs (n = 24)

TMF title (year)	Describe intra- and/or inter-organizational networks?	Characterize ties (e.g., by strength or homophily)?	Characterize aspects of network structure (e.g., centrality, density)?
Research Development and Dissemination Framework (1969) [51]	Intra and inter	X	X
Real-world Dissemination (1992) [54]	Intra and inter		X
Convergent Diffusion and Social Marketing Approach to Dissemination (1996) [41,42]	Intra and inter	X	X
Sticky Knowledge (1996) [46,59]	Intra only	X	
Model for Locally-based Research Transfer Development (1999) [37]	Inter only		
Research-to-Practice Framework (2000) [58]	Inter only		X
Framework for the Dissemination and Utilization of Research for Health Care Policy and Practice (2002) [43,44]	Intra and inter		X
Conceptualizing Dissemination Research and Activity: Canadian Heart Health Initiative (2003) [45,55]	Inter only		
Diffusion of Innovations (2003) [21]	Intra and inter	X	X
Exposure, Experience, Expertise, Embedding (“4E”) Framework (2003) [36,47]	Intra and inter		
Framework for Knowledge Translation (2003) [52]	Intra and inter	X	X
Conceptual Model for the Diffusion of Innovations in Service Organizations (2004) [26]	Intra and inter	X	X
Availability, Responsiveness & Continuity (ARC): An Organizational Community Intervention Model (2005) [49]	Intra and inter		X
Implementation Research Framework (2005) [48]	Intra and inter		
Linking Systems (2005) [56]	Intra and inter	X	X
Pathways to Evidence-informed Policy and Practice (2005) [38]	Intra and inter		X
Replicating Effective Programs (2007) [32]	Intra and inter		
Stages of Research Utilization Model (2007) [40]	Intra and inter	X	X
Framework of Dissemination in Health Services Intervention Research (2008) [53]	Intra and inter	X	X
Consolidated Framework for Implementation Research (2009) [29,39]	Intra and inter	X	X
Blueprint for Dissemination (2010) [35]	Intra and inter		X
Exploration, Preparation, Implementation, Sustainment (EPIS) Framework (2011) [23,24]	Intra and inter	X	
Approach/Engagement, Implementation, Monitoring, Sustainability (AIMS) Model (2014) [57]	Intra and inter	X	X
Community-based Learning Collaborative Model (2016) [50,60]	Intra and inter	X	X

TMFs = theories, models, and frameworks.

*Note:* TMFs are listed in ascending order by year first published. Further details about each framework are available in Supplementary File 3.

### Tie type and purpose

Network function varied across TMFs, but commonly included the flow of resources, knowledge, trust, norms, goals, and values to influence the dissemination and/or implementation of innovations. For example, the Stages of Research Utilization Model [40] emphasizes the exchange of information and knowledge about the program innovation between the “resource system” consisting of innovation developers and the “user system” consisting of implementing organizations. Additionally, several frameworks – including the AIMS (Approach/Engagement, Implementation, Monitoring, Sustainability) model [57], the CFIR [29,39], and the Framework of Dissemination in Health Services Intervention Research [53] – discussed how networks build collective efficacy for D&I efforts by empowering network members and fostering a sense of community.

### Tie characteristics

Approximately half of the network-explicit TMFs (n = 13; 54%) described factors that characterize ties or relationships (i.e., qualifying information beyond the existence of ties) (Table 3). For example, Diffusion of Innovations [21] and the AIMS model [57] make the distinction between strong and weak ties. Strong ties made of trusting relationships are important for providing social support, whereas weak ties may be better suited for spreading novel information [9]. Several TMFs described tie strength based on the level of trust between network members. For example, the Framework for Knowledge Translation posits that knowledge transfer between the research and user groups will be more successful if their relationships have a higher level of trust, rapport, and prior history of working together [52]. Many TMFs [21,26,29,39,41,42,50,60] also use the concept of homophily (or conversely, heterophily) in characterizing ties based on the similarity (or difference) of connected actors according to a given characteristic [61]. In these TMFs, homophilous ties based on concordant occupation, professional role, and/or cultural background may facilitate the adoption of innovations.

### Network structural characteristics

Most network-explicit TMFs (n = 17; 71%) characterized element(s) of network structure, such as size, centrality, density, and bridging (Table 3). Related to the role of networks in dissemination strategies, the Blueprint for Dissemination emphasizes (a) the importance of network size in generating a threshold of participating organizations to maximize information exchange, and (b) a “nodal structure” consisting of a centralized organizing body at the national level paired with local organizations to drive progress in dissemination [35]. The Conceptual Model for the Diffusion of Innovations in Service Organizations discusses network structure at length, including the distinction between decentralized, horizontal networks that are helpful for informal diffusion versus centralized, vertical networks that are often needed for active dissemination [26]. Related to network density, the Convergent Diffusion and Social Marketing Approach to Dissemination describes the difference between cohesive, interconnected “ecologic communities” made up of local “cooperating and competing organizations” versus the less integrated network structure of “societal sectors” (e.g., government agencies, non-profit organizations) [41,42]. Several TMFs emphasize the specific role of actors as “bridges” across groups or “boundary spanners,” including those that connect the inner setting where implementation occurs with external organizations in the outer setting [23,24,26,49,56]. For example, a key network feature of the Linking Systems framework is the presence of a “linking agent” who brokers knowledge and connects the gap between innovation developers and users [56].

### Network change interventions

From most TMFs, we could infer at least one approach to changing the structure or characteristics of networks involved in D&I efforts (n = 21; 88%). Following Valente’s categorization of network change interventions [10], the most common network change approach was *identifying individuals –* for example, opinion leaders, innovators, early adopters, program or organizational champions, change agents, coaches, bridges, and knowledge brokers – to lead or support D&I efforts (n = 20) [21,23,24,26,29,32,35,36,38–42,45,47–57,60]. Several TMFs also described network change approaches related to segmentation (n = 10) [21,23,24,26,29,32,35,39,41,42,51,54,56], induction (n = 8) [21,23,24,26,29,35,39,41,42,50,54,60], and alteration (n = 11) [23,24,35,37,45,48,50–52,54–57,60]. *Segmentation*, for example, is described in the Replicating Effective Programs framework with the intentional tailoring of intervention approaches and dissemination efforts for different recipient populations and implementing organizations [32]. Considerations of *induction* are often related to the deliberate spread of innovations or information throughout a network. This approach is exemplified in the Blueprint for Dissemination, which offers guidance for sharing best practices among peer organizations in a learning network and “generating a threshold of participating organizations that maximizes network” [35]. Network *alteration* approaches included the creation of inter-organizational relationships, for example, through building coalitions [35,45,55] or “Community Change Teams” as described in the Community-based Learning Collaborative Model [50,60]. Changes to network structure were also discussed in the context of knowledge translation or brokerage by establishing new ties to exchange information across groups or communities [52,56].

## Discussion

This review examined the presence and presentation of core social network concepts in community-level D&I TMFs to identify opportunities to study and leverage social networks in D&I science. Among 66 relevant TMFs, we found that about one-third (n = 24; 36%) explicitly attended to social networks, and among those that did, there was an emphasis on network change involving individual actors. Our examination of network-explicit TMFs highlights opportunities to think more broadly about network change interventions and outcomes. Readers interested in applying network concepts in D&I research and practice may use the results of our review to help select appropriate TMFs to guide network-related inquiry in several areas or entry points. This may include considerations of existing organizational structures or groups (e.g., coalitions, community advisory boards, learning collaboratives, government), network features or characteristics (e.g., power and hierarchy, common language and values, organizational context and culture, relationships within and across sectors), and outcomes of network activity on practice and policy (e.g., knowledge translation, capacity building, fostering trust, provision of technical assistance).

There is interest in D&I science to incorporate perspectives of whole implementation systems and affect systems change among organizations and communities involved in disseminating and implementing EBIs [7,17,20,63]. Our findings highlight several D&I TMFs focused on community-level change that give explicit attention to networks, which can support opportunities for intervention. For example, the systems-oriented nature of the EPIS framework – which focuses on a range of actors (including policymakers, funders, advocacy groups, and payors) and inter-organizational networks (including contractual links or academic–community partnerships) – offers a strong foundation for guiding network change in support of pre-implementation, implementation, and sustainment phases [23,24]. As a result, empirical projects grounded in EPIS have examined and intervened on social networks to support the adoption and high-quality implementation of EBIs [12]. Building on EPIS, the Community-based Learning Collaborative Model [50,60] provides additional guidance on specific network characteristics and change processes throughout various stages of implementation. D&I researchers aiming to understand implementation determinants from a network and systems perspective may consider the Updated CFIR published in 2022, with attention to constructs delineating actors’ roles, network connections between the inner and outer setting, and “teaming” processes to collaboratively implement an innovation [39]. The results of this work do not suggest that a broad range of TMFs are inappropriate for use in D&I science. Instead, a large number may benefit from expansion to incorporate a network perspective. Of course, as noted by Nilsen, combining theoretical concepts requires careful consideration of the underlying philosophies and worldviews represented to ensure coherence in the integrated framework [64].

As noted earlier, a range of network change interventions may be important potential implementation strategies, such as building coalitions [5,10]. However, the observed emphasis on network change strategies focused on individual behavior change points to the need to think more broadly about network-based D&I strategies. The focus on individual-level strategies is unsurprising given the well-documented roles of opinion leaders [65] and champions [66], among other change agents, in the D&I literature. This finding also aligns with that of a 2023 review of network change strategies for implementation, which also observed a high prevalence of individual-level network targets [18]. That work highlighted the potential for change in the network context and the network environment to reshape systems supporting community-level efforts to implement or disseminate EBIs [18]. This broadened focus beyond individual action may also help advance equity by shifting expectations of who is involved and/or at the center of D&I networks. Leveraging existing networks of partners, such as community coalitions [19,20] and professional learning communities [67], may also provide an important foundation to promote equity. By considering implementation systems more holistically, D&I scientists can take advantage of the potential for D&I efforts to not only support the spread of a given innovation through a system but also reshape that system to be more equity-promoting [68].

We found that most network-explicit TMFs included a dual focus on ties within and across organizations. This emphasizes the theoretical importance of understanding and considering intra- and inter-organizational relationships for D&I research and how such diversity of tie structure may influence implementation outcomes. For example, with a focus on building social capital for D&I efforts, the Implementation Capital Framework published in 2019 [69] highlights the impact of high bonding social capital (i.e., strong within-group ties and clustering) on sharing norms related to an EBI’s acceptability and appropriateness. This could be highly relevant for intra-organizational connections, as an example. At the same time, the framework emphasizes how high levels of bridging social capital (i.e., strong between-group ties) can afford actors access to diverse information and resources and impact an EBI’s feasibility and fidelity.

Another important insight from this review is the need for future research to explore the dynamic (and malleable) nature of social networks. Thus, after network change interventions are deployed, changes in social networks could serve as an important implementation outcome. To further this line of inquiry, clear specification of the purpose and expected impact of network-focused implementation strategies is needed. Measurement will also be strengthened by pairing network theory with existing SNA tools that describe, visualize, and model network changes over time [7]. There are increasing opportunities for researchers to harness SNA tools through the growth of open science/data and open-source initiatives and platforms [70,71]. The push for publicly available research data means that a growing numbers of social network datasets and analysis programming code can serve as roadmaps for researchers to conduct SNA. Likewise, growing knowledge in open-source programming languages, such as R and Python, opens to door for social network experts to collaborate on packages specifically designed to aid in SNA [72]. This extends to open-source tools like Gephi Lite which allow for a user-friendly web-based interface to upload and interact with their social network data [73]. These technological advances drive the feasibility for researchers to incorporate and evaluate network-focused implementation strategies.

We also acknowledge that the quality, relevance, and impact of SNA efforts in D&I science are likely to be much higher if a diverse range of actors are involved throughout [7,68,74]. Further details about participatory SNA work in D&I are available elsewhere [7]; however, connected to participatory work is understanding social networks in the context of advancing health equity. There are a wide range of opportunities, from increasing inclusion of diverse expertise through strategic partnership composition [75] to seeking transformational change to reduce inequities for the focal condition as well as others [68]. Future research is needed to explore these opportunities further.

In sum, our review offers several important research opportunities for SNA in D&I science: improving examination of networks as determinants; further developing strategies for network change and network-focused implementation strategies; advancing use of network measures to understand the impact of such network change strategies; and broadening our understanding of how networks can help advance health equity. Advancing D&I science in these areas may benefit from participatory approaches that engage diverse community partners representing the real-world settings and contexts in which the D&I of EBIs occur [68]. Partnerships with community-based organizations, public health departments, and local policymakers, for example, can inform (a) the section and potential tailoring of appropriate TMF(s) to guide network-related inquiry and practice, and (b) the selection, tailoring, and testing of network-focused implementation strategies on equity-focused outcomes related to dissemination, implementation, and community health.

This narrative review has important limitations. First, our findings are based on community-level TMFs included in the Pinto et al. sample of TMFs [3]. While this sampling frame is appropriate given our focus on community-level change for disseminating and/or implementing EBIs, other relevant TMFs may have been excluded from the Pinto et al. review, and by extension, our review. Another source of potential TMFs for interested readers is the “Dissemination & Implementation Models in Health” interactive webtool (which includes a community-level filter) [76]. This repository builds on the Tabak et al. 2012 review of D&I TMFs [77], which was a key data source for the Pinto et al. article. Second, our review included TMFs originally published over the past several decades, and given our sampling approach, we may have excluded more recently developed TMFs. However, older TMFs represent the important history and multidisciplinary foundations of D&I science. Many older TMFs remain highly salient by guiding current empirical D&I work, the development of new TMFs, and ongoing TMF refinement. Third, our review included articles that presented TMFs and not the range of applications that may include additional detail regarding networks’ roles in the dissemination or implementation of specific EBIs. However, the study’s goal was to examine the guidance offered generally through TMF descriptions (which other researchers can draw upon); thus, the pool of included data sources seemed appropriate. Fourth, as with any narrative review, the data reflect what team members were and were not able to parse from the TMFs as originally presented. We minimized the risk of omission or miscoding by utilizing dual independent coding, having a third consensus reviewer, and team discussions.

At the same time, the strengths of this review offer important benefits. We provide a novel network lens to a large set of community-level D&I TMFs. Our documentation of network characteristics in these TMFs may help D&I researchers identify entry points and opportunities for integrating network perspectives in their work. We also identify key areas for future SNA and D&I research to help purposively guide the field moving forward.

## Conclusions

Our findings highlight several community-level D&I TMFs that emphasize networks. Among frameworks with an explicit network focus, the variability of network actors and activities suggests an opportunity to advance the understanding of diverse social drivers of D&I processes. Further research is needed to examine networks (at multiple levels) as determinants, network change interventions as implementation strategies, and/or network change as an implementation outcome in community-level D&I efforts.

## Supporting information

Korn et al. supplementary material 1 Korn et al. supplementary material

Korn et al. supplementary material 2 Korn et al. supplementary material

Korn et al. supplementary material 3 Korn et al. supplementary material

## Supplementary material

The supplementary material for this article can be found at https://doi.org/10.1017/cts.2024.614.

## References

[1] Brownson RC , Kumanyika SK , Kreuter MW , Haire-Joshu D. Implementation science should give higher priority to health equity. Implement Sci. 2021;16(1):28. doi: 10.1186/s13012-021-01097-0. 33740999 PMC7977499

[2] Brownson RC , Colditz GA , Proctor EK. Dissemination and Implementation Research in Health: Translating Science to Practice. 3rd ed. New York, NY: Oxford University Press; 2023.

[3] Pinto RM , (Ethan) Park S , Miles R , Ong PN. Community engagement in dissemination and implementation models: a narrative review. Implement Res Pract. 2021;2:263348952098530. doi: 10.1177/2633489520985305. PMC9978697 37089998

[4] Robert Wood Johnson Foundation. A new way to talk about the social determinants of health. Robert Wood Johnson Foundation, 2010. (https://www.rwjf.org/en/library/research/2010/01/a-new-way-to-talk-about-the-social-determinants-of-health.html) Accessed November 12, 2024.

[5] Powell BJ , Waltz TJ , Chinman MJ , et al. A refined compilation of implementation strategies: results from the expert recommendations for implementing change (ERIC) project. Implement Sci. 2015;10(1):21. doi: 10.1186/s13012-015-0209-1. 25889199 PMC4328074

[6] Leeman J , Birken SA , Powell BJ , Rohweder C , Shea CM. Beyond “implementation strategies”: classifying the full range of strategies used in implementation science and practice. Implement Sci. 2017;12(1):125. doi: 10.1186/s13012-017-0657-x. 29100551 PMC5670723

[7] Luke DA , Morshed AB , McKay VR , Combs TB. Systems science methods in dissemination and implementation research. In: Brownson RC , Colditz GA , Proctor EK , eds. Dissemination and Implementation Research in Health: Translating Science to Practice. 3rd ed. Oxford University Press; 2023:269–288.

[8] Burke JG , Lich KH , Neal JW , Meissner HI , Yonas M , Mabry PL. Enhancing dissemination and implementation research using systems science methods. Int J Behav Med. 2015;22(3):283–291. doi: 10.1007/s12529-014-9417-3. 24852184 PMC4363012

[9] Light R , Moody J , eds. The Oxford Handbook of Social Networks. New York, NY: Oxford University Press; 2020.

[10] Valente TW. Network interventions. Science. 2012;337(6090):49–53. doi: 10.1126/science.1217330. 22767921

[11] Glegg SMN , Jenkins E , Kothari A. How the study of networks informs knowledge translation and implementation: a scoping review. Implement Sci. 2019;14(1):34. doi: 10.1186/s13012-019-0879-1. 30917844 PMC6437864

[12] Palinkas LA , Holloway IW , Rice E , Fuentes D , Wu Q , Chamberlain P. Social networks and implementation of evidence-based practices in public youth-serving systems: a mixed-methods study. Implement Sci. 2011;6(1):113. doi: 10.1186/1748-5908-6-113. 21958674 PMC3216853

[13] Luke DA , Powell BJ , Paniagua-Avila A. Bridges and mechanisms: integrating systems science thinking into implementation research. Annu Rev Public Health. 2024;45(1):7–25. doi: 10.1146/annurev-publhealth-060922-040205. 38100647

[14] Luke DA , Harris JK. Network analysis in public health: history, methods, and applications. Annu Rev Public Health. 2007;28(1):69-02–93. doi: 10.1146/annurev.publhealth.28.021406.144132. 17222078

[15] Hunter RF , de la Haye K , Murray JM , et al. Social network interventions for health behaviours and outcomes: a systematic review and meta-analysis. PLOS Med. 2019;16(9):e1002890. doi: 10.1371/journal.pmed.1002890. 31479454 PMC6719831

[16] Shelton RC , Lee M , Brotzman LE , et al. Use of social network analysis in the development, dissemination, implementation, and sustainability of health behavior interventions for adults: a systematic review. Soc Sci Med 1982. 2019;220:81–101. doi: 10.1016/j.socscimed.2018.10.013. PMC7857673 30412922

[17] Gesell SB , Barkin SL , Valente TW. Social network diagnostics: a tool for monitoring group interventions. Implement Sci. 2013;8(1):116. doi: 10.1186/1748-5908-8-116. 24083343 PMC3851809

[18] Bunger AC , Yousefi-Nooraie R , Warren K , Cao Q , Dadgostar P , Bustos TE. Developing a typology of network alteration strategies for implementation: a scoping review and iterative synthesis. Implement Sci. 2023;18(1):10. doi: 10.1186/s13012-023-01266-3. 37024916 PMC10080780

[19] Provan KG , Veazie MA , Staten LK , Teufel-Shone NI. The use of network analysis to strengthen community partnerships. Public Adm Rev. 2005;65(5):603–613. doi: 10.1111/j.1540-6210.2005.00487.x.

[20] Valente TW , Chou CP , Pentz MA. Community coalitions as a system: effects of network change on adoption of evidence-based substance abuse prevention. Am J Public Health. 2007;97(5):880–886. doi: 10.2105/AJPH.2005.063644. 17329667 PMC1854884

[21] Rogers EM. Diffusion of Innovations. New York, NY: Simon and Schuster; 2003.

[22] Covidence, https://www.covidence.org/, Accessed October 17, 2022.

[23] Aarons GA , Hurlburt M , Horwitz SM. Advancing a conceptual model of evidence-based practice implementation in public service sectors. Adm Policy Ment Health Ment Health Serv Res. 2011;38(1):4–23. 10.1007/s10488-010-0327-7 PMC3025110 21197565

[24] Moullin JC , Dickson KS , Stadnick NA , Rabin B , Aarons GA. Systematic review of the exploration, preparation, implementation, sustainment (EPIS) framework. Implement Sci. 2019;14(1):1. doi: 10.1186/s13012-018-0842-6. 30611302 PMC6321673

[25] Wandersman A , Duffy J , Flaspohler P , et al. Bridging the gap between prevention research and practice: the interactive systems framework for dissemination and implementation. Am J Community Psychol. 2008;41(3-4):171–181. doi: 10.1007/s10464-008-9174-z. 18302018

[26] Greenhalgh T , Robert G , Macfarlane F , Bate P , Kyriakidou O. Diffusion of innovations in service organizations: systematic review and recommendations. Milbank Q. 2004;82(4):581–629. doi: 10.1111/j.0887-378X.2004.00325.x. 15595944 PMC2690184

[27] Chambers DA , Glasgow RE , Stange KC. The dynamic sustainability framework: addressing the paradox of sustainment amid ongoing change. Implement Sci. 2013;8(1):117. doi: 10.1186/1748-5908-8-117. 24088228 PMC3852739

[28] Shelton RC , Chambers DA , Glasgow RE. An extension of RE-AIM to enhance sustainability: addressing dynamic context and promoting health equity over time. Front Public Health. 2020;8(134):134. doi: 10.3389/fpubh.2020.00134. 32478025 PMC7235159

[29] Damschroder LJ , Aron DC , Keith RE , Kirsh SR , Alexander JA , Lowery JC. Fostering implementation of health services research findings into practice: a consolidated framework for advancing implementation science. Implement Sci. 2009;4(1):50. doi: 10.1186/1748-5908-4-50. 19664226 PMC2736161

[30] Woodward EN , Matthieu MM , Uchendu US , Rogal S , Kirchner JE. The health equity implementation framework: proposal and preliminary study of hepatitis C virus treatment. Implement Sci. 2019;14(1):26. doi: 10.1186/s13012-019-0861-y. 30866982 PMC6417278

[31] Michie S , van Stralen MM , West R. The behaviour change wheel: a new method for characterising and designing behaviour change interventions. Implement Sci. 2011;6(1):42. doi: 10.1186/1748-5908-6-42. 21513547 PMC3096582

[32] Kilbourne AM , Neumann MS , Pincus HA , Bauer MS , Stall R. Implementing evidence-based interventions in health care: application of the replicating effective programs framework. Implement Sci. 2007;2(1):42. doi: 10.1186/1748-5908-2-42. 18067681 PMC2248206

[33] Raghavan R , Bright CL , Shadoin AL. Toward a policy ecology of implementation of evidence-based practices in public mental health settings. Implement Sci. 2008;3(1):26. doi: 10.1186/1748-5908-3-26. 18485219 PMC2396668

[34] Atun R , de Jongh T , Secci F , Ohiri K , Adeyi O. Integration of targeted health interventions into health systems: a conceptual framework for analysis. Health Policy Plan. 2010;25(2):104–111. doi: 10.1093/heapol/czp055. 19917651

[35] Yuan CT , Nembhard IM , Stern AF , Brush JE Jr , Krumholz HM , Bradley EH. Blueprint for the dissemination of evidence-based practices in health care. Issue Brief Commonw Fund. 2010;86:1–16. 20469542

[36] Farkas M , Jette AM , Tennstedt S , Haley SM , Quinn V. Knowledge dissemination and utilization in gerontology: an organizing framework. Gerontologist. 2003;1(suppl_1):47–56. doi: 10.1093/geront/43.suppl_1.47. 12637689

[37] Anderson M , Cosby J , Swan B , Moore H , Broekhoven M. The use of research in local health service agencies. Soc Sci Med. 1999;49(8):1007–1019. doi: 10.1016/S0277-9536(99)00179-3. 10475666

[38] Bowen S , Zwi AB. Pathways to, evidence-informed, policy and practice: a framework for action. PLoS Med. 2005;2(7):e166. doi: 10.1371/journal.pmed. 15913387 PMC1140676

[39] Damschroder LJ , Reardon CM , Widerquist MAO , Lowery J. The updated consolidated framework for implementation research based on user feedback. Implement Sci. 2022;17(1):75. doi: 10.1186/s13012-022-01245-0. 36309746 PMC9617234

[40] Davis SM , Peterson JC , Helfrich CD , Cunningham-Sabo L. Introduction and conceptual model for utilization of prevention research. Am J Prev Med. 2007;33(1 Suppl):S1–S5. doi: 10.1016/j.amepre.2007.04.004. 17584588

[41] Dearing JW , Maibach EW , Buller DB. A convergent diffusion and social marketing approach for disseminating proven approaches to physical activity promotion. Am J Prev Med. 2006;31(4 Suppl):S11–23. doi: 10.1016/j.amepre.2006.06.018. 16979466

[42] Dearing JW , Rogers EM , Meyer G , et al. Social marketing and diffusion-based strategies for communicating with unique populations: HIV prevention in San Francisco. J Health Commun. 1996;1(4):343–363. doi: 10.1080/108107396127997. 10947368

[43] Dobbins M , DeCorby K , Robeson P , Tirilis D , Rycroft-Malone J , Bucknall T. Public health model. Models Framew Implement Evid-Based Pract Link Evid Action. 2010; 2:268.

[44] Dobbins M , Ciliska D , Cockerill R , Barnsley J , DiCenso A. A framework for the dissemination and utilization of research for health-care policy and practice. Online J Knowl Synth Nurs. 2002;9(1):7–160. doi: 10.1111/j.1524-475X.2002.00149.x. 12439759

[45] Elliott SJ , O’Loughlin J , Robinson K , et al. Conceptualizing dissemination research and activity: the case of the Canadian heart health initiative. Health Educ Behav. 2003;30(3):267–282. discussion 283 doi: 10.1177/1090198103030003003. 19731496

[46] Elwyn G , Taubert M , Kowalczuk J. Sticky knowledge: a possible model for investigating implementation in healthcare contexts. Implement Sci. 2007;2(1):44. doi: 10.1186/1748-5908-2-44. 18096040 PMC2231385

[47] Farkas M , Anthony WA. Bridging science to service: using rehabilitation research and training center program to ensure that research-based knowledge makes a difference. J Rehabil Res Dev. 2007;44(6):879–892. doi: 10.1682/Jrrd.2006.08.0101. 18075944

[48] Fixsen DL , Naoom SF , Blase KA , et al. *Implementation research: a synthesis of the literature*. University of South Florida, Louis de la Parte Florida Mental Health Institute. The National Implementation Research Network. 2005. Louis de la Parte Florida Mental Health Institute NIRN-MonographFull-01-2005.pdf.

[49] Glisson C , Schoenwald SK. The ARC organizational and community intervention strategy for implementing evidence-based children’s mental health treatments. Ment Health Serv Res. 2005;7(4):243–259. doi: 10.1007/s11020-005-7456-1. 16320107

[50] Hanson RF , Saunders BE , Ralston E , Moreland AD , Peer SO , Fitzgerald MM. Statewide implementation of child trauma-focused practices using the community-based learning collaborative model. Psychol Serv. 2019;16(1):170–181. doi: 10.1037/ser0000319. 30550316 PMC6361698

[51] Havelock RG. Planning for Innovation through Dissemination and Utilization of Knowledge. Center for Research on Utilization of Scientific Knowledge, Institute for Social Research. Ann Arbor, Michigan: University of Michigan; 1969.

[52] Jacobson N , Butterill D , Goering P. Development of a framework for knowledge translation: understanding user context. J Health Serv Res Policy. 2003;8(2):94–99. doi: 10.1258/135581903321466067. 12820671

[53] Mendel P , Meredith LS , Schoenbaum M , Sherbourne CD , Wells KB. Interventions in organizational and community context: a framework for building evidence on dissemination and implementation in health services research. Adm Policy Ment Health. 2008;35(1-2):21–37. doi: 10.1007/s10488-007-0144-9. 17990095 PMC3582701

[54] Pettigrew A , Ferlie E , McKee L. Shaping Strategic Change. London: Sage; 1992.

[55] Riley BL , Stachenko S , Wilson E , et al. Can the Canadian heart health initiative inform the population health intervention research initiative for Canada? Can J Public Health. 2009;100(1):I20–I26. 10.1007/BF03405505 PMC6973562 19263979

[56] Robinson K , Elliott SJ , Driedger SM , et al. Using linking systems to build capacity and enhance dissemination in heart health promotion: a Canadian multiple-case study. Health Educ Res. 2004;20(5):499–513. doi: 10.1093/her/cyh006. 15613492

[57] Smith EP , Wise E , Rosen H , Rosen A , Childs S , McManus M. Top-down, bottom-up, and around the jungle gym: a social exchange and networks approach to engaging afterschool programs in implementing evidence-based practices. Am J Community Psychol. 2014;53(3-4):491–502. doi: 10.1007/s10464-014-9656-0. 24781678 PMC4634874

[58] Sogolow ED , Kay LS , Doll LS , et al. Strengthening HIV prevention: application of a research-to-practice framework. AIDS Educ Prev. 2000;12(5 Suppl):21–32. 11063067

[59] Szulanski G. Exploring internal stickiness: impediments to the transfer of best practice within the firm. Strateg Manag J. 1996;17(S2):27–43. doi: 10.1002/smj.4250171105.

[60] Hanson RF , Schoenwald S , Saunders BE , et al. Testing the community-based learning collaborative (CBLC) implementation model: a study protocol. Int J Ment Health Syst. 2016;10(1):52. doi: 10.1186/s13033-016-0084-4. 27547240 PMC4991101

[61] McPherson JM , Smith-Lovin L , Cook JM. Birds of a feather: homophily in social networks. Annu Rev Sociol. 2001;27(1):415–444.

[62] Dearing JW , Beacom AM , Chamberlain SA , et al. Pathways for best practice diffusion: the structure of informal relationships in Canada’s long-term care sector. Implement Sci IS. 2017;12(1):11. doi: 10.1186/s13012-017-0542-7. 28159009 PMC5291985

[63] Valente TW , Palinkas LA , Czaja S , Chu KH , Brown CH. Social Network Analysis for Program Implementation. PLOS ONE. 2015;10(6):e0131712. doi: 10.1371/journal.pone.0131712. 26110842 PMC4482437

[64] Nilsen P. Overview of theories, models and frameworks in implementation science. In: Nilsen P , Birken SA , eds. Handbook on Implementation Science. Edward Elgar Publishing; 2020, doi: 10.4337/9781788975995.00008.

[65] Flodgren G , Parmelli E , Doumit G , et al. Local opinion leaders: effects on professional practice and health care outcomes. Cochrane Database Syst Rev. 2011; (8): CD000125. doi: 10.1002/14651858.CD000125.pub4. 21833939 PMC4172331

[66] Miech EJ , Rattray NA , Flanagan ME , Damschroder L , Schmid AA , Damush TM. Inside help: an integrative review of champions in healthcare-related implementation. SAGE Open Med. 2018;6:2050312118773261. doi: 10.1177/2050312118773261. 29796266 PMC5960847

[67] Bunger AC , Hanson RF , Doogan NJ , Powell BJ , Cao Y , Dunn J. Can learning collaboratives support implementation by rewiring professional networks? Adm Policy Ment Health Ment Health Serv Res. 2016;43(1):79–92. doi: 10.1007/s10488-014-0621-x. PMC4485606 25542237

[68] Ramanadhan S , Aleman R , Bradley C , et al. Using participatory implementation science to advance health equity. Annu Rev Public Health. 2024;45(1):47–67. doi: 10.1146/annurev-publhealth-060722-024251. 38109515 PMC11251496

[69] Neal JW , Neal ZP. Implementation capital: merging frameworks of implementation outcomes and social capital to support the use of evidence-based practices. Implement Sci. 2019;14(1):16. doi: 10.1186/s13012-019-0860-z. 30764850 PMC6376677

[70] National Institutes of Health. NOT-OD-21-013: Final NIH Policy for Data Management and Sharing. 2020. https://grants.nih.gov/grants/guide/notice-files/NOT-OD-21-013.html, Accessed July 1, 2024.

[71] Center for Open Science. Center for Open Science: 2023 Impact Report. 2023. https://www.cos.io/impact, Accessed July 1, 2024.

[72] Csárdi G , Nepusz T , Müller K , et al. igraph for R: r interface of the igraph library for graph theory and network analysis. Published online February 20, 2024;20:768–2609. doi: 10.5281/ZENODO.7682609.

[73] Lite Gephi. 2024. https://gephi.org/gephi-lite/, Accessed July 1, 2024.

[74] Carothers BJ , Allen P , Walsh-Bailey C , et al. Mapping the lay of the land: using interactive network analytic tools for collaboration in rural cancer prevention and control. Cancer Epidemiol Biomarkers Prev. 2022;31(6):1159–1167. doi: 10.1158/1055-9965.EPI-21-1446. 35443033 PMC9167755

[75] Ramanadhan S , Salhi C , Achille E , et al. Addressing cancer disparities via community network mobilization and intersectoral partnerships: a social network analysis. PLoS ONE. 2012;7(2):e32130. doi: 10.1371/journal.pone.0032130. 22384156 PMC3285642

[76] Dissemination & Implementation Models in Health 2024. https://dissemination-implementation.org/, Accessed March 29, 2024.

[77] Tabak RG , Khoong EC , Chambers DA , Brownson RC. Bridging research and practice: models for dissemination and implementation research. Am J Prev Med. 2012;43(3):337–350. doi: 10.1016/j.amepre.2012.05.024. 22898128 PMC3592983

[78] Winkler JD , Lohr KN , Brook RH. Persuasive communication and medical technology assessment. Arch Intern Med. 1985;145(2):314–317. 3977492

[79] Funk SG , Tornquist EM , Champagne MT. A model for improving the dissemination of nursing research. West J Nurs Res. 1989;11(3):361–372. doi: 10.1177/019394598901100311. 2750148

[80] Lester JP. The utilization of policy analysis by state agency officials. Knowl-Creat Diffus Util. 1993;14(3):267–290. doi: 10.1177/107554709301400301.

[81] Pathman DE , Konrad TR , Freed GL , Freeman VA , Koch GG. The awareness-to-adherence model of the steps to clinical guideline compliance. The case of pediatric vaccine recommendations. Med Care. 1996;34(9):873–889. doi: 10.1097/00005650-199609000-00002. 8792778

[82] Green L , Kreuter M. Health Program Planning: An Educational and Ecological Approach. New York: McGraw-Hill; 2005.

[83] Davis D , Evans M , Jadad A , et al. The case for knowledge translation: shortening the journey from evidence to effect. Bmj. 2003;327(7405):33–35. doi: 10.1136/bmj.327.7405.33. 12842955 PMC164240

[84] Orlandi MA. Health promotion technology transfer: organizational perspectives. Can J Public Health. 1996;87:(Suppl 2), S28–33. 9002340

[85] Logan J , Graham ID. Toward a comprehensive interdisciplinary model of health care research use. Sci Commun. 1998;20(2):227–246. doi: 10.1177/1075547098020002004.

[86] Logan J , Graham ID . The Ottawa Model of Research Use. In Models and frameworks for implementing evidence-based practice. Oxford: Wiley-Blackwell, 2010:83–108.

[87] Martin GW , Herie MA , Turner BJ , Cunningham JA. A social marketing model for disseminating research-based treatments to addictions treatment providers. Addiction. 1998;93(11):1703–1715. doi: 10.1046/j.1360-0443.1998.931117038.x. 9926533

[88] Kitson A , Harvey G , McCormack B. Enabling the implementation of evidence based practice: a conceptual framework. Qual Health Care. 1998;7(3):149–158. doi: 10.1136/qshc.7.3.149. 10185141 PMC2483604

[89] Kitson AL , Rycroft-Malone J , Harvey G , McCormack B , Seers K , Titchen A. Evaluating the successful implementation of evidence into practice using the PARiHS framework: theoretical and practical challenges. Implement Sci. 2008;3(1):1. doi: 10.1186/1748-5908-3-1. 18179688 PMC2235887

[90] Rycroft-Malone J. The PARIHS framework - a framework for guiding the implementation of evidence-based practice. J Nurs Care Qual. 2004;19(4):297–304. doi: 10.1097/00001786-200410000-00002. 15535533

[91] Glasgow RE , Vogt TM , Boles SM. Evaluating the public health impact of health promotion interventions: the RE-AIM framework. Am J Public Health. 1999;89(9):1322–1327. doi: 10.2105/ajph.89.9.1322. 10474547 PMC1508772

[92] Kraft JM , Mezoff JS , Sogolow ED , Neumann MS , Thomas PA. A technology transfer model for effective HIV/AIDS interventions: science and practice. AIDS Educ Prev. 2000;12(5 Suppl):7–20. 11063066

[93] Scullion PA. Effective dissemination strategies. Nurse Res. 2002;10(1):65–77. doi: 10.7748/nr2002.10.10.1.65.c5880. 12405007

[94] Cooper LA , Hill MN , Powe NR. Designing and evaluating interventions to eliminate racial and ethnic disparities in health care. J Gen Intern Med. 2002;17(6):477–486. doi: 10.1046/j.1525-1497.2002.10633.x. 12133164 PMC1495065

[95] Reardon R , Lavis J , Gibson J. From Research to Practice: A Knowledge Transfer Planning Guide. Toronto, Ontario: Institute for Work & Health; 2006.

[96] Ellen ME , Lavis JN , Ouimet M , Grimshaw J , Bedard PO. Determining research knowledge infrastructure for healthcare systems: a qualitative study. Implement Sci. 2011;6(1):60. doi: 10.1186/1748-5908-6-60. 21645401 PMC3123231

[97] Lavis JN , Lomas J , Hamid M , Sewankambo NK. Assessing country-level efforts to link research to action. Bull World Health Organ. 2006;84(8):620–628. doi: 10.2471/blt.06.030312. 16917649 PMC2627430

[98] Lavis JN , Robertson D , Woodside JM , McLeod CB , Abelson J , Knowledge Transfer Study G. How can research organizations more effectively transfer research knowledge to decision makers? Milbank Q. 2003;81(2):221–248, 171. doi: 10.1111/1468-0009.t01-1-00052. 12841049 PMC2690219

[99] Swinburn B , Gill T , Kumanyika S. Obesity prevention: a proposed framework for translating evidence into action. Obes Rev. 2005;6(1):23–33. doi: 10.1111/j.1467-789X.2005.00184.x. 15655036

[100] Michie S , Johnston M , Abraham C , et al. Making psychological theory useful for implementing evidence based practice: a consensus approach. Qual Saf Health Care. 2005;14(1):26–33. doi: 10.1136/qshc.2004.011155. 15692000 PMC1743963

[101] Kilbourne AM , Switzer G , Hyman K , Crowley-Matoka M , Fine MJ. Advancing health disparities research within the health care system: a conceptual framework. Am J Public Health. 2006;96(12):2113–2121. doi: 10.2105/AJPH.2005.077628. 17077411 PMC1698151

[102] Owen N , Glanz K , Sallis JF , Kelder SH. Evidence-based approaches to dissemination and diffusion of physical activity interventions. Am J Prev Med. 2006;31(4 Suppl):S35–44. doi: 10.1016/j.amepre.2006.06.008. 16979468

[103] Green LW , Orleans CT , Ottoson JM , Cameron R , Pierce JP , Bettinghaus EP. Inferring strategies for disseminating physical activity policies, programs, and practices from the successes of tobacco control. Am J Prev Med. 2006;31(4 Suppl):S66–81. doi: 10.1016/j.amepre.2006.06.023. 16979471

[104] Bauman AE , Nelson DE , Pratt M , Matsudo V , Schoeppe S. Dissemination of physical activity evidence, programs, policies, and surveillance in the international public health arena. Am J Prev Med. 2006;31(4 Suppl):S57–65. doi: 10.1016/j.amepre.2006.06.026. 16979470

[105] Atun RA , Kyratsis I , Jelic G , Rados-Malicbegovic D , Gurol-Urganci I. Diffusion of complex health innovations--implementation of primary health care reforms in Bosnia and Herzegovina. Health Policy Plan. 2007;22(1):28–39. doi: 10.1093/heapol/czl031. 17237492

[106] Tolson D , Booth J , Lowndes A. Achieving evidence-based nursing practice: impact of the caledonian development model. J Nurs Manag. 2008;16(6):682–691. doi: 10.1111/j.1365-2834.2008.00889.x. 18808462

[107] Pronovost PJ , Berenholtz SM , Needham DM. Translating evidence into practice: a model for large scale knowledge translation. Bmj. 2008;337:a1714. doi: 10.1136/bmj.a1714. 18838424

[108] Fleming E , Perkins J , Easa D , et al. The role of translational research in advancing health disparities: a conceptual framework. J Ethn Dis. 2008;18(2):S2-155-60. PMC2705204 18646340

[109] Ward V , House A , Hamer S. Developing a framework for transferring knowledge into action: a thematic analysis of the literature. J Health Serv Res Policy. 2009;14(3):156–164. doi: 10.1258/jhsrp.2009.008120. 19541874 PMC2933505

[110] Ward VL , House AO , Hamer S. Knowledge brokering: exploring the process of transferring knowledge into action. BMC Health Serv Res. 2009;9(1):1–6. 19149888 10.1186/1472-6963-9-12 PMC2632997

[111] Ward V , Smith S , House A , Hamer S. Exploring knowledge exchange: a useful framework for practice and policy. Soc Sci Med. 2012;74(3):297–304. doi: 10.1016/j.socscimed.2011.09.021. 22014420

[112] Murray E , Treweek S , Pope C , et al. Normalisation process theory: a framework for developing, evaluating and implementing complex interventions. BMC Med. 2010;8(1):63. doi: 10.1186/1741-7015-8-63. 20961442 PMC2978112

[113] Green LW , Ottoson JM , Garcia C , Hiatt RA. Diffusion theory and knowledge dissemination, utilization, and integration in public health. Annu Rev Public Health. 2009;30(1):151-03–174. doi: 10.1146/annurev.publhealth.031308.100049. 19705558

[114] Proctor EK , Landsverk J , Aarons G , Chambers D , Glisson C , Mittman B. Implementation research in mental health services: an emerging science with conceptual, methodological, and training challenges. Adm Policy Ment Health. 2009;36(1):24–34. doi: 10.1007/s10488-008-0197-4. 19104929 PMC3808121

[115] Ogilvie D , Craig P , Griffin S , Macintyre S , Wareham NJ. A translational framework for public health research. BMC Public Health. 2009;9(1):116. doi: 10.1186/1471-2458-9-116. 19400941 PMC2681470

[116] TIDIRH Working Group. Interacting elements of integrating science, policy, and practice. Training institute for dissemination and implementation research in health. Conference proceedings, Chapel Hill NC, 2011.

[117] Harris JR , Cheadle A , Hannon PA , et al. A framework for disseminating evidence-based health promotion practices. Prev Chronic Dis. 2012;9:E22. 22172189 PMC3277406

[118] Dreisinger ML , Boland EM , Filler CD , Baker EA , Hessel AS , Brownson RC. Contextual factors influencing readiness for dissemination of obesity prevention programs and policies. Health Educ Res. 2012;27(2):292–306. doi: 10.1093/her/cyr063. 21893684

[119] Kreuter M , Casey C , Bernhardt J , Brownson R , Colditz G , Proctor E. Enhancing dissemination through marketing and distribution systems: a vision for public health. In: Ross C. Brownson, Graham A. Colditz, Enola K Proctor, eds. Dissemination and Implementation Research in Health: Translating Science to Practice. New York, NY: Oxford University Press, 2012; 213–222.

[120] Dodson E , Brownson R , Weiss S , Brownson R , Colditz G , Proctor E. Policy dissemination research. In: Ross C. Brownson, Graham A. Colditz, and Enola K. Proctor (eds), Dissemination and Implementation Research in Health: Translating Science to Practice. Published Online, 1st edn, Oxford Academic, 2012. 10.1093/acprof:oso/9780199751877.003.0021 Accessed November 15, 2024.

[121] Glasgow RE , Green LW , Taylor MV , Stange KC. An evidence integration triangle for aligning science with policy and practice. Am J Prev Med. 2012;42(6):646–654. doi: 10.1016/j.amepre.2012.02.016. 22608384 PMC4457385

[122] Neta G , Glasgow RE , Carpenter CR , et al. A framework for enhancing the value of research for dissemination and implementation. Am J Public Health 2015;105(1):49–57. doi: 10.2105/AJPH.2014.302206. 25393182 PMC4265905

[123] Redmond LC , Jock B , Gadhoke P , et al. OPREVENT (Obesity prevention and evaluation of interVention effectiveness in naTive north Americans): design of a multilevel, multicomponent obesity intervention for native American adults and households. Curr Dev Nutr. 2019;3(Suppl 2):81–93. doi: 10.1093/cdn/nzz009. 31453430 PMC6700458

[124] Barnett M , Miranda J , Kia-Keating M , Saldana L , Landsverk J , Lau AS. Developing and evaluating a lay health worker delivered implementation intervention to decrease engagement disparities in behavioural parent training: a mixed methods study protocol. BMJ Open 2019;9(7):e028988. doi: 10.1136/bmjopen-2019-028988. PMC6661633 31324682

[125] Kingdon JW . Agendas, Alternatives, and Public Policies, vol. 45. Boston: Little, Brown; 1984.

[126] Kingdon JW. Agendas, Alternatives, and Public Policies. 2nd edn, Boston: Pearson; 2010.

[127] Israel BA , Schulz AJ , Parker EA , Becker AB. Review of community-based research: assessing partnership approaches to improve public health. Annu Rev Public Health. 1998;19(1):173–202. doi: 10.1146/annurev.publhealth.19.1.173. 9611617

[128] Bartholomew LK , Parcel GS , Kok G. Intervention mapping: a process for developing theory- and evidence-based health education programs. Health Educ Behav. 1998;25(5):545–563. doi: 10.1177/109019819802500502. 9768376

[129] Nolan K , Schall MW , Erb F , Nolan T. Using a framework for spread: the case of patient access in the veterans health administration. Jt Comm J Qual Patient Saf. 2005;31(6):339–347. doi: 10.1016/S1553-7250(05)31045-2. 15999964

[130] Langley GJ , Moen RD , Nolan KM , Nolan TW , Norman CL , Provost LP. The Improvement Guide: A Practical Approach to Enhancing Organizational Performance. San Francisco: Jossey-Bass: 2009.

[131] Damush T , Bravata D , Plue L , Woodward-Hagg H , William L. Facilitation of best practices (FAB) framework: stroke QUERI center annual report. Am J Prev Med. 2008;43:337–350.

[132] Rutten A , Wolff A , Streber A. Sustainable implementation of evidence-based programmes in health promotion: a theoretical framework and concept of interactive knowledge to action. Gesundheitswesen Bundesverb Arzte Offentlichen Gesundheitsdienstes Ger. 2016;78(3):139–145. doi: 10.1055/s-0035-1548883. 25985226

