# Supplementary material for: Advancing and strengthening the study of social networks in community-level dissemination and implementation research: A narrative review
Source: J Clin Transl Sci. 2024 Oct 28;8(1):e203. doi: 10.1017/cts.2024.614 (PMC11626584; doi:10.1017/cts.2024.614)
Supplement: Korn et al. supplementary material 1 — Korn et al. supplementary material [file S2059866124006149sup001.docx]

**Supplementary File 1.** List of theories, models, and frameworks (TMFs) included (n=66) and excluded (n=8) in the narrative review based on the Pinto et al. sample of 74 TMFs^3^

| **TMF title (year)** |
| --- |
| ***Network-explicit TMFs (n=24)*** |
| 1. Research Development and Dissemination Framework (1969)^51^ |
| 1. Real-world Dissemination (1992)^54^ |
| 1. Convergent Diffusion and Social Marketing Approach to Dissemination (1996)^42,62^ |
| 1. Sticky Knowledge (1996)^46,59^ |
| 1. Model for Locally-based Research Transfer Development (1999)^37^ |
| 1. Research-to-Practice Framework (2000)^58^ |
| 1. Framework for the Dissemination and Utilization of Research for Health Care Policy and Practice (2002)^43,44^ |
| 1. Conceptualizing Dissemination Research and Activity: Canadian Heart Health Initiative (2003)^45,55^ |
| 1. Diffusion of Innovations (2003)^21^ |
| 1. Exposure, Experience, Expertise, Embedding (“4E”) Framework (2003)^36,47^ |
| 1. Framework for Knowledge Translation (2003)^52^ |
| 1. Conceptual Model for the Diffusion of Innovations in Service Organizations (2004)^26^ |
| 1. Availability, Responsiveness & Continuity (ARC): An Organizational Community Intervention Model (2005)^49^ |
| 1. Implementation Research Framework (2005)^48^ |
| 1. Linking Systems (2005)^56^ |
| 1. Pathways to Evidence-informed Policy and Practice (2005)^38^ |
| 1. Replicating Effective Programs (2007)^32^ |
| 1. Stages of Research Utilization Model (2007)^40^ |
| 1. Framework of Dissemination in Health Services Intervention Research (2008)^53^ |
| 1. Consolidated Framework for Implementation Research (2009)^29,39^ |
| 1. Blueprint for Dissemination (2010)^35^ |
| 1. Exploration, Preparation, Implementation, Sustainment (EPIS) Framework (2011)^23,24^ |
| 1. Approach/Engagement, Implementation, Monitoring, Sustainability (AIMS) Model (2014)^57^ |
| 1. Community-based Learning Collaborative Model (2016)^50,60^ |
| ***TMFs that are not network-explicit (n=42)*** |
| 1. RAND Model of Persuasive Communication and Diffusion of Medical Innovation (1985)^78^ |
| 1. Model for Improving the Dissemination of Nursing Research (1989)^79^ |
| 1. Conceptual Model of Knowledge Utilization (1993)^80^ |
| 1. Awareness-to-Adherence Model (1996)^81–83^ |
| 1. Health Promotion Technology Transfer (1996)^84^ |
| 1. Ottawa Model of Research Use (OMRU) (1998)^85,86^ |
| 1. Outpatient Treatment in Ontario Services (OPTIONS) Model (1998)^87^ |
| 1. Promoting Action on Research Implementation in Health Services (PARIHS) (1998)^88–90^ |
| 1. RE-AIM (1999)^91^ |
| 1. Technology Transfer Model (2000)^92^ |
| 1. Effective Dissemination Strategies (2002)^93^ |
| 1. Designing and Evaluating Interventions to Eliminate Racial and Ethnic Disparities in Health Care (2002)^94^ |
| 1. Research Knowledge Infrastructure (2003)^95–98^ |
| 1. Framework for Translating Evidence into Action (2005)^99^ |
| 1. Explaining Behavior Change in Evidence-based Practice (2005)^100^ |
| 1. Advancing Health Disparities Research within the Health Care System (2006)^101^ |
| 1. Policy Framework for Diffusion of Evidence Based Physical Activity Interventions (2006)^102^ |
| 1. Push-Pull Capacity Model (2006)^103^ |
| 1. Six-Step Framework for International Physical Activity Dissemination (2006)^104^ |
| 1. Framework for Analyzing the Diffusion of Complex Innovations in Health Systems (2007)^34,105^ |
| 1. Policy Ecology of Implementation Framework (2008)^33^ |
| 1. Interactive Systems Framework (ISF) (2008)^25^ |
| 1. Caledonian Development Model (2008)^106^ |
| 1. Pronovost's 4E Process Theory (2008)^107^ |
| 1. Translational Research Framework to Address Health Disparities (2008)^108^ |
| 1. Knowledge Transfer Framework (2009)^109–111^ |
| 1. Normalisation Process Theory (2009)^112^ |
| 1. Utilization-Focused Surveillance Framework (2009)^113^ |
| 1. Conceptual Model of Implementation Research (2009)^114^ |
| 1. Translational Framework for Public Health Research (2009)^115^ |
| 1. Interacting Elements of Integrating Science, Policy, And Practice (2011)^116^ |
| 1. Behavior Change Wheel (2011)^31^ |
| 1. Health Promotion Research Center Framework (2012)^117^ |
| 1. Dissemination of Evidence-Based Interventions to Prevent Obesity (2012)^118^ |
| 1. Marketing and Distribution System for Public Health (2012)^119^ |
| 1. Framework for Dissemination of Evidence-based Policy (2012)^120^ |
| 1. Evidence Integration Triangle (EIT) Model (2012)^121^ |
| 1. Dynamic Sustainability Framework (2013)^27^ |
| 1. Framework for Enhancing the Value of Research for Dissemination and Implementation (2015)^122^ |
| 1. Health Equity Implementation Framework (2019)^30^ |
| 1. Obesity Prevention and Evaluation of InterVention Effectiveness in NaTive North Americans (OPREVENT) (2019)^123^ |
| 1. Lay Health Workers Enhancing Engagement for Parents (LEEP) (2019)^124^ |
| ***TMFs excluded from Pinto et al. list (n=8) and reasons for exclusion*** |
| 1. Streams of Policy Process (1984);^125,126^ out of scope |
| 1. Community-based Participatory Research (CBPR) (1998);^127^ out of scope (not a D&I TMF) |
| 1. Intervention Mapping (1998);^128^ out of scope (focused on intervention development) |
| 1. PRECEDE-PROCEED model (2005);^82^ out of scope |
| 1. Framework for Spread (2005);^129,130^ out of scope |
| 1. Facilitating Adoption of Best Practices (FAB) Model (2008);^131^ full text not available |
| 1. Interactive Knowledge to Action Framework (2016);^132^ not available in English |
| 1. Community-based Learning Collaborative Model (2016);^60^ duplicate (merged with ^50^) |

*Abbreviations*. TMFs: theories, models, and frameworks.
