# Supplementary material for: Advancing and strengthening the study of social networks in community-level dissemination and implementation research: A narrative review
Source: J Clin Transl Sci. 2024 Oct 28;8(1):e203. doi: 10.1017/cts.2024.614 (PMC11626584; doi:10.1017/cts.2024.614)
Supplement: Korn et al. supplementary material 2 — Korn et al. supplementary material [file S2059866124006149sup002.pdf]

Preview

#1. TMF title

If the TMF name is not obvious, can cross-reference with the TMF names listed in Table 2 of the Pinto et al. (2021) review:

https://journals.sagepub.com/doi/full/10.1177/2633489520985305#_i24

#2. Year TMF first published

If extracting from multiple records, select the earliest publication year.

#3. Are networks explicitly addressed in the TMF?

Select "yes" if the TMF explicitly addresses networks as a construct, the structure of relationships, functional aspects of the network (e.g., social capital), and/or what is exchanged across the network (e.g., the innovation, resources, influence, norms, advice). If "no", describe what network perspectives can be inferred from the TMF in #4 below (if applicable).

1. Yes
2. No

#4. What "network-adjacent" perspectives can be inferred from the TMF?

Give the range of network-adjacent concepts. This might include considerations of communication, social influence (e.g., behavioral modeling, peer influence), and/or implementation processes that can contribute to network thinking. If network perspectives cannot be inferred, report "not applicable". Coders may respond to this question in addition to selecting "Yes" in #3 above.

#5. What types of actors are referenced in the TMF?

Select all that apply. Think about the actor's role or what they are doing to support implementation.

1. No actors specified
2. Recipients, patients, or clients (i.e., people getting the health service of interest)
3. Client interpersonal network members (e.g., family members or peers)
4. Providers, practitioners, service-delivery persons, implementers
5. Implementation support professionals (e.g., technical assistance providers)
6. Managers
7. Adopters or decision makers (e.g., organizational leaders)
8. Policy makers
9. Funders or payors
10. Researchers or innovation developers
11. Implementing or disseminating agencies or organizations
12. Community-based organizations (CBOs) or faith-based organizations (FBOs)
13. Other

#6. Are networks within (intra) or across (inter) organizations?

Select all that apply.

1. Not applicable
2. Not specified
3. Intra-organizational
4. Inter-organizational

5. ☐ Other

#7. Are networks in the inner and/or outer setting or context?

Select all that apply. “Not applicable” should be selected for dissemination frameworks and TMFs without a network focus. Inner setting is where the implementation occurs. Outer setting includes other organizations and external influences beyond where implementation occurs.

1. ☐ Not applicable
2. ☐ Not specified (i.e., too vague to tell)
3. ☐ Inner setting
4. ☐ Outer setting

#8. What is built or exchanged across the network(s) and/or the outcome(s) of network activity?

Describe what is built or exchanged across individuals or groups (e.g., organizations) that make up the network as an indication of network function and/or the outcome(s) of network activity. This might include the innovation, ideas, knowledge, information, resources, influence, values or norms, goals, social support, advice, trust, social cohesion, social capital, collective efficacy etc. Otherwise report “not applicable” or “not specified”.

#9. How are relationships (or ties) characterized?

Describe how the TMF addresses the strength or weakness of ties (e.g., frequency of contact, level of trust) or other characteristics like tie homophily (similarity of connected actors based on a given attribute). Otherwise report “not applicable” or “not specified”.

#10. How is network structure considered?

Describe aspects of network structure that are addressed in the TMF. This might include considerations of network size, centrality (connectedness), centralization or hierarchy, density, transitivity (triads or network closure), etc.* Otherwise report “not applicable” or “not specified”.

*the following citations may be helpful:

Valente TW. Network interventions. Science (New York, NY). 2012;337(6090):49-53. doi:10.1126/science.1217330

Glegg SMN, Jenkins E, Kothari A. How the study of networks informs knowledge translation and implementation: a scoping review. Implementation Science. 2019;14(1):34. doi:10.1186/s13012-019-0879-1

#11. How does the TMF propose ways to intervene on or change the network?

If applicable, coders may use Valente's categories of "network interventions"* (defined below) as a guide to describe network changes or network interventions. If so, indicate the appropriate category in addition to a description (e.g., "Individual identification: identify a program champion at each organization"). It is okay to copy and paste text from the article; if so, be sure to use quotations.

Coders are encouraged to be generous and make inferences as applicable. It is okay if the network intervention or network change does not fit within one of the pre-defined Valente categories.*

*Individual identification: identifying individuals to act as change agents (e.g., opinion leaders, champions, bridges, those on the network periphery)

*Segmentation: delivering an intervention to segments or subgroups in a network (e.g., cliques)

*Induction: inducing or prompting change using network structure (e.g., word-of-mouth tactics, snowballing)

*Alteration: changing the network structure itself by adding or removing individuals, groups, or ties (sometimes called "rewiring")

*Valente TW. Network interventions. Science (New York, NY). 2012;337(6090):49-53. doi:10.1126/science.1217330

#12. Other comments

Describe anything else not captured previously related to networks. If the TMF has multiple versions that differ in their network focus, also describe those changes here.

#13. Is this TMF a good example of how to think about networks in D&I, and if so, how?

Describe what is interesting about the TMF from a network perspective. This information will be helpful for the paper's discussion section. Otherwise report "not applicable".

Feedback & Support

Find your answer, fast.

We've got a new Knowledge base that answers many frequently asked questions. Why not try that first?

View the knowledge base OR Send us an email
