# Supplementary material for: Advancing and strengthening the study of social networks in community-level dissemination and implementation research: A narrative review
Source: J Clin Transl Sci. 2024 Oct 28;8(1):e203. doi: 10.1017/cts.2024.614 (PMC11626584; doi:10.1017/cts.2024.614)
Supplement: Korn et al. supplementary material 3 — Korn et al. supplementary material [file S2059866124006149sup003.docx]

**Supplementary File 3.** Detailed characteristics of network-explicit theories, models, and frameworks (TMFs) (n=24)

| **TMF title (year)** | **Nodes** | **Ties** | **Tie type and purpose** | **Tie characteristics** | **Structural characteristics** | **Network change interventions**^10^ |
| --- | --- | --- | --- | --- | --- | --- |
| Research Development and Dissemination Framework (1969)^51^ | innovation recipients; implementers; implementation support professionals; adopters or decision makers; policy makers; researchers or innovation developers; implementing or disseminating agencies or orgs.; community-based or faith-based orgs.; other: media | intra- and inter-org. networks | knowledge flows into the organization, is consumed, and then flows out; exchange of norms, values, and trust | homophily | group cohesiveness; hierarchy of org. subunits; centralization and decentralization; centrality | individual identification; segmentation; alteration |
| Real-world Dissemination (1992)^54^ | implementers; adopters or decision makers; implementing or disseminating agencies or orgs.; community-based or faith-based orgs. | intra- and inter-org. networks | exchange of perceived benefits of innovation; networks yield social cohesion, improved implementation processes and buy-in | not specified | boundary spanning | individual identification; segmentation; induction; alteration |
| Convergent Diffusion and Social Marketing Approach to Dissemination (1996)^41,42^ | innovation recipients; client interpersonal network members; implementers; implementation support professionals; adopters or decision makers; policy makers; researchers or innovation developers; implementing or disseminating agencies or orgs.; community-based or faith-based orgs. | intra- and inter-org. networks | communication flows to inform adoption and adaptation of evidence-based practices and programs | homophily | density of cooperating and competing organizations; centrality | individual identification; segmentation; induction |
| Sticky Knowledge (1996)^46,59^ | implementers; adopters or decision makers; implementing or disseminating agencies or orgs.; other: employees within firm/organization | intra-org. networks only | exchange of information, knowledge, and resources about organizational best practices | level of tie trust and credibility | not specified | not specified |
| Model for Locally-based Research Transfer Development (1999)^37^ | innovation recipients; implementers; adopters or decision makers; policy makers; researchers or innovation developers; implementing or disseminating agencies or orgs.; community-based or faith-based orgs. | inter-org. networks only | research transfer and information sharing (influenced by bi-directional awareness, communication, and interactions between community-based decision-makers, their agencies, and researchers) | not specified | not specified | alteration |
| Research-to-Practice Framework (2000)^58^ | implementers; policy makers; funders or payors; researchers or innovation developers; implementing or disseminating agencies or orgs. | inter-org. networks only | information exchange; technology transfer; collaboration; planning, priority setting, and technical assistance | not specified | centralization | not specified |
| Framework for the Dissemination and Utilization of Research for Health Care Policy and Practice (2002)^43,44^ | implementers; adopters or decision makers; policy makers; researchers or innovation developers; implementing or disseminating agencies or orgs.; community-based or faith-based orgs. | intra- and inter-org. networks | exchange of health policy and practice innovations (evidence-based practices); collaboration and competition | not specified | centralization of decision-makers; organizational hierarchy and vertical differentiation | not specified |
| Conceptualizing Dissemination Research and Activity: Canadian Heart Health Initiative (2003)^45,55^ | adopters or decision makers; researchers or innovation developers; implementing or disseminating agencies or orgs.; community-based or faith-based orgs. | inter-org. networks only | exchange of resources, innovative solutions, technical support, peer networking, and feedback | not specified | not specified | individual identification; alteration |
| Diffusion of Innovations (2003)^21^ | innovation recipients; client interpersonal network members; implementers; implementation support professionals; adopters or decision makers; researchers or innovation developers | intra- and inter-org. networks | exchange of information and advice about innovations, technological solutions, and knowledge, values, and norms | homophily and heterophily; strength of weak ties | centrality; bridging; centralization and decentralization; density; network size; org. subunits | individual identification; segmentation; induction |
| Exposure, Experience, Expertise, Embedding (“4E”) Framework (2003)^36,47^ | innovation recipients; client interpersonal network members; implementers; implementation support professionals; adopters or decision makers; policy makers; funders or payors; researchers or innovation developers; implementing or disseminating agencies or orgs.; community-based or faith-based orgs. | intra- and inter-org. networks | exchange of knowledge, information, expertise, support, and positive attitudes; networks facilitate peer support, mentorship, and role modeling | not specified | not specified | individual identification |
| Framework for Knowledge Translation (2003)^52^ | adopters or decision makers; policy makers; researchers or innovation developers; other: advocacy groups | intra- and inter-org. networks | exchange of knowledge and information from research group to user group; information flow within user group | level of trust and rapport; communication frequency; homophily; tie stability among user group | network size; centralization | individual identification; alteration |
| Conceptual Model for the Diffusion of Innovations in Service Organizations (2004)^26^ | innovation recipients; implementers; implementation support professionals; adopters or decision makers; researchers or innovation developers; implementing or disseminating agencies or orgs. | intra- and inter-org. networks | exchange of innovation and shared meaning, values, and goals; flow of knowledge and resources; networks facilitate social support, training, and technical assistance | homophily | centralization vs. decentralization; horizontal vs. vertical structure; centrality; density; “fuzzy boundaries” with a “hard core and soft periphery” | individual identification; segmentation; induction |
| Availability, Responsiveness & Continuity (ARC): An Organizational Community Intervention Model (2005)^49^ | adopters or decision makers; client interpersonal network members; implementers; community-based or faith-based orgs. | intra- and inter-org. networks | exchange of information, trust, feedback, and technical assistance regarding intervention; fostering shared understanding or norms; participatory decision-making; social support and social cohesion | not specified | boundary spanning and bridging | individual identification |
| Implementation Research Framework (2005)^48^ | innovation recipients; implementers; implementation support professionals; adopters or decision makers; researchers or innovation developers; implementing or disseminating agencies or orgs.; community-based or faith-based orgs. | intra- and inter-org. networks | flow of information, knowledge, and resources; sharing of org. norms and values; enabling support for the innovation and performance feedback between practitioners and program implementers | not specified | not specified | individual identification; alteration |
| Linking Systems (2005)^56^ | innovation recipients; implementers; implementation support professionals; adopters or decision makers; policy makers; researchers or innovation developers | intra- and inter-org. networks | exchange of knowledge, ideas, resources, practices; enabling social support and capacity building; collaboration; shared understanding of an org. or program's needs, abilities, and issues | bi-directional relationships; communication frequency; heterophily between diverse groups | extent of centralization; bridging role of linking agent between user and resources groups | individual identification; segmentation; alteration |
| Pathways to Evidence-informed Policy and Practice (2005)^38^ | implementers; adopters or decision makers; policy makers; researchers or innovation developers; implementing or disseminating agencies or orgs.; other: advocacy groups | intra- and inter-org. networks | mutual exchange of ideas, knowledge, and experience; networks facilitate adoption and diffusion of evidence into policy and practice | not specified | centrality and connectedness; organizational hierarchy and centralization | individual identification |
| Replicating Effective Programs (2007)^32^ | innovation recipients; implementers; implementation support professionals; researchers or innovation developers; implementing or disseminating agencies or orgs.; community-based or faith-based orgs. | intra- and inter-org. networks | knowledge sharing; collaborative decision-making; fostering buy-in; providing technical assistance and feedback | not specified | not specified | individual identification; segmentation |
| Stages of Research Utilization Model (2007)^40^ | innovation recipients; implementers; implementation support professionals; adopters or decision makers; policy makers; funders or payors; researchers or innovation developers; implementing or disseminating agencies or orgs.; community-based or faith-based orgs. | intra- and inter-org. networks | exchanging information and knowledge about the innovation between the “resource system” (researchers and developers) and the "user system" (implementing orgs. and agencies) | tie strength; quality and frequency of communication | bridging structure via a “linking agent” between the resource and user systems | individual identification |
| Framework of Dissemination in Health Services Intervention Research (2008)^53^ | innovation recipients; client interpersonal network members; implementers; implementation support professionals; adopters or decision makers; funders or payors; implementing or disseminating agencies or orgs.; community-based or faith-based orgs. | intra- and inter-org. networks | flow of information, knowledge, and resources; sharing norms, beliefs, and values; building trust, social support, and collective efficacy | level of mutual trust; extent of shared beliefs and org. climate; homophily | centrality; role of peripheral actors | individual identification |
| Consolidated Framework for Implementation Research (2009)^29,39^ | innovation recipients; implementers; implementation support professionals; adopters or decision makers; implementing or disseminating agencies or orgs.; community-based or faith-based orgs. | intra- and inter-org. networks | exchange of information, knowledge, and influence; building social capital and collective efficacy | tie strength, formality, and tangibility; homophily | clustering; network size; centralization | individual identification; segmentation; induction |
| Blueprint for Dissemination (2010)^35^ | implementers; adopters or decision makers; policy makers; funders or payors; researchers or innovation developers; implementing or disseminating agencies or orgs. | intra- and inter-org. networks | exchange of information, best practices, and advice; spread of social norms and pressure; mentorship | not specified | network size; centralization and hierarchy of nodal org. structure | individual identification; segmentation; induction; alteration |
| Exploration, Preparation, Implementation, Sustainment (EPIS) Framework (2011)^23,24^ | implementers; implementation support professionals; adopters or decision makers; researchers or innovation developers; implementing or disseminating agencies or orgs.; community-based or faith-based orgs. | intra- and inter-org. networks | flow of information, resources, and influence; sharing of values and goals; facilitates provision of implementation support; transmitting culture and climate | homophily and heterophily | not specified | individual identification; segmentation; induction; alteration |
| Approach/Engagement, Implementation, Monitoring, Sustainability (AIMS) Model (2014)^57^ | implementers; implementation support professionals; adopters or decision makers; researchers or innovation developers; implementing or disseminating agencies or orgs.; community-based or faith-based orgs. | intra- and inter-org. networks | flow of knowledge, information, and resources; building collective efficacy, “sense of community and power”, social support, and trust | bi-directional trust; tie strength and weakness; interaction frequency | centralization | individual identification; alteration |
| Community-based Learning Collaborative Model (2016)^50,60^ | implementers; implementation support professionals; adopters or decision makers; implementing or disseminating agencies or orgs.; community-based or faith-based orgs. | intra- and inter-org. networks | flow of information, knowledge, and resources; client coordination and referrals; goal sharing and consensus building; cultivation of mutual trust, social cohesion, and social capital; collaboration | homophily and heterophily; level of trust and relationship satisfaction | network size; density; network sub-units; centrality; hierarchy; brokerage structure for referrals | individual identification; induction; alteration |

*Note*. Theories, models, and frameworks (TMFs) are listed in ascending order by year first published.
